# Companion animals and mental health: a narrative synthesis of how pets deliver therapeutic mechanisms outside formal provision

**DOI:** 10.3389/fpsyt.2026.1803383

**Published:** 2026-07-01

**Authors:** Emily Vicary, Anne Rogers, Helen Brooks

**Affiliations:** 1 Division of Nursing, Midwifery and Social Work, School of Health Sciences, Manchester Academic Health Centre, University of Manchester, Manchester, United Kingdom; 2 Faculty of Health Sciences, University of Southampton, Southampton, United Kingdom

**Keywords:** companion animals, emotional regulation, mental health, psychological therapy, qualitative synthesis, therapeutic mechanisms

## Abstract

**Background:**

Evidence highlighting the emotional and social support that companion animals provide for people managing mental health problems has increased in recent years. However, the therapeutic mechanisms underpinning these benefits remain poorly understood. This qualitative review aimed to explore these perceived mechanisms in the context of established psychosocial models and therapeutic approaches commonly used in formal mental health provision.

**Methods:**

A framework and line of argument synthesis were used to analyse the 24 studies included in the narrative review. Eligible studies were those that presented primary qualitative data exploring relationships with companion animals and the impact of these relationships on owners’ mental health and emotional distress. Extracted data were mapped onto established clinical and psychosocial therapeutic approaches, enabling the identification of mechanisms operating across contexts and their sequencing.

**Results:**

Key mechanisms identified were emotional co-regulation, safety, and trust. These relational conditions created a foundation which supported the organisation and pacing of daily life and strengthened individuals’ sense of agency through the development of routine and responsibility. These processes facilitated identity reconstruction and sense-making, contributing to the perceived therapeutic value of companion animals in managing mental health and emotional distress.

**Conclusion:**

The review suggests that the therapeutic value of companion animal support operates through a layered, relational process that differs markedly from those offered by formal interventions. The perceived therapeutic advantage of companion animals arises from their alignment with everyday human needs, needs that formal provision struggles to consistently meet. Pets make visible the limitations of professionalised, episodic care and underscore the importance of continuity, non-judgment, and embodied presence.

## 1 Introduction

Mental health problems represent a growing global burden and have significant impacts on individuals, communities and health systems (1). Despite the availability of evidence-based psychological interventions, many people who meet diagnostic criteria do not access formal mental health services due to limited service capacity, stigma and misalignment of provision with personal preferences (2). As a result, there is growing interest in understanding how informal, relational, activity and community-based inputs might support mental health and wellbeing in ways that complement, support, or substitute for engagement with formal therapeutic input. Developing this understanding and theorising the mechanisms through which non-formal support systems operate is likely to be relevant to contemporary mental health policy and practice environments and models of therapy (3).

Research on companion animals and mental health has expanded substantially, although the wider evidence base remains mixed. Quantitative studies have reported positive, negative, and null associations between pet ownership and wellbeing, suggesting that ownership status alone is unlikely to explain mental health outcomes (4–6). Qualitative evidence, however, more consistently indicates that people living with mental health difficulties perceive companion animals as important sources of emotional, social, and practical support (7, 8). This distinction is important because it shifts analytic attention from whether pet ownership is associated with better mental health at population level to how particular human-animal relationships are experienced as supportive in everyday life. These accounts position companion animals as relational figures embedded in daily routines, sometimes comparable to family members or close social network ties, and describe support through companionship, emotional closeness, reciprocity, routine, and social connection. Unlike many forms of human social support, relationships with pets are typically characterised by constancy, availability, and embeddedness in everyday routines and shared activities. Thus, companion animals occupy a distinctive position within the informal support landscapes of people living with mental health difficulties and long-term conditions more generally. Companion animal relationships are present across diverse groups of people and life stages and are embedded within a range of social, cultural and socioeconomic contexts (8, 9). Interactions also typically occur outside of formal therapeutic environments and encounters. As a result, they represent an important opportunity for research on understanding a set of relationships and therapeutic mechanisms that might contribute to mental health management.

Theoretically, companion animal support sits at the intersection of relational, regulatory, and recovery-oriented accounts of mental health. Social buffering and attachment perspectives suggest that distress is moderated not only through explicit advice or problem-solving, but through perceived availability of a trusted other, proximity during threat, and felt security within emotionally salient relationships (10, 11). Within human-animal relationships, these processes may be intensified by forms of non-verbal presence, tactile contact, and perceived non-judgment that do not depend on disclosure or reflective insight. Brown’s account of companion animals as “selfobjects” is useful here because it positions pets as relational figures through which continuity of self, emotional containment, and felt recognition may be sustained (12). More recent relational processes similarly caution against treating wellbeing as an individual outcome produced by ownership alone, instead emphasising how human and animal lives become mutually organised through everyday practices of care, routine, and co-presence (13). These perspectives suggest that the therapeutic value of companion animals may lie less in discrete “effects” of pet ownership than in the relational conditions through which regulation, agency, identity, and meaning become possible.

The present review focuses on perceived therapeutic mechanisms identifiable within participants’ qualitative accounts. Therapeutic mechanisms are understood here as psychosocial and relational processes through which companion animals are described as helping people manage distress, maintain functioning, or sustain a sense of self in everyday life. These include, for example, emotional co-regulation, felt safety, non-judgement, routine, responsibility, social connection, identity reconstruction, and meaning-making. A mechanism-focused synthesis enables an integrated understanding of how companion animal support is perceived to operate, how these processes align with or diverge from formal therapeutic approaches, and how mechanisms may accumulate or sequence over time. Rather than treating benefits as isolated outcomes, this approach consolidates fragmented qualitative findings into a coherent explanatory framework with relevance for policy, service design, and intervention development.

In current policy and practice contexts, companion animal support is most often positioned as informal, adjunctive, or peripheral to formal mental health provision, rather than as a source of therapeutic processes requiring analysis in its own right (8). This is not because animals have been absent from therapeutic research. Animal-assisted interventions have generated a substantial literature, including studies examining structured programmes delivered in clinical, educational, or community settings (14, 15). However, this intervention literature differs from the focus of the present review. Our concern is with owned companion animals and the everyday relationships through which people living with mental health difficulties perceive support to occur outside formal provision. The gap addressed here, therefore, is not whether animals can be incorporated into professional intervention, but how qualitative research on ordinary companion animal relationships can illuminate therapeutic mechanisms enacted through daily life, relational continuity, and non-professional care.

While formal mental health interventions delivered by trained professionals differ in substantive respects from support provided by companion animals – most notably in relation to interpretation, clinical expertise, and the use of structured therapeutic models – the mechanisms through which therapies exert their effects may not be entirely exclusive to professional practice. Core therapeutic processes such as emotional regulation, attachment, routine, non-judgmental presence, and embodied interaction are also prominent features of human-animal relationships (7, 16). Some scholars argue that the boundaries often drawn between human and non-human capacities for understanding and communication may be overstated, highlighting substantial evidence that human-animal interactions are complex, reciprocal, and relational and that these relationships confer significant physical and psychological benefits (17, 18).

Previous reviews, including work by the authors, suggest that companion animals are perceived to confer benefits for people experiencing mental health difficulties (8). This qualitative emphasis is important in the context of a wider quantitative literature that remains mixed (5, 6), because it shifts attention from whether pet ownership is associated with mental health outcomes at population level to how particular human–animal relationships are experienced as supportive, under what conditions, and through which perceived therapeutic processes. There remains a need for a more focused and theoretically informed synthesis of qualitative evidence that examines how these perceived benefits are generated, for whom, under what conditions, and how they relate to established therapeutic frameworks. Addressing this gap is particularly important in the context of increasing pressure on mental health services, workforce shortages, and policy interest in non-clinical, community-based, and preventative approaches to mental health support.

This narrative review therefore aims to synthesise qualitative research exploring the mechanisms and outcomes of companion animal engagement among people living with mental health difficulties. By aligning themes from extant literature with recognised therapeutic approaches, the review seeks to inform applied health services research, policy development, and service design, and to contribute to debates about the potential role of companion animals within contemporary mental health support systems. Specifically, we sought to answer the following questions: *how do people living with mental health difficulties describe companion animals as supporting emotional distress, daily functioning, and sense of self; what perceived therapeutic mechanisms can be identified across these qualitative accounts*; and *what do these accounts suggest about forms of support that may be difficult to provide through time-limited, verbally mediated, professionally delivered interventions?*

## 2 Methods

### 2.1 Study design

This study builds on *a priori* systematic review of evidence on companion animals and mental health (8). The current study re-analysed qualitative studies from that review and conducted updated searches to identify additional relevant studies published since 2017. A narrative review, with a framework and line-of-argument synthesis, was conducted to examine qualitative evidence on how relationships with companion animals are described as contributing to mental health across diverse populations (19, 20).

This review was designed as a narrative review and interpretive qualitative synthesis rather than as a *de novo* systematic review. Its purpose was not to provide exhaustive evidence surveillance, effect estimation, or formal update of all records retrieved in the original systematic review, but to develop a theoretically informed synthesis of perceived therapeutic mechanisms across qualitative studies of companion animal relationships and mental health. Accordingly, the search strategy was designed to identify additional conceptually relevant qualitative studies published since the previous review, while analytic emphasis was placed on interpretation, comparison, and theory development across eligible studies.

A narrative synthesis approach enabled interpretive engagement with heterogeneous qualitative findings, while a framework-based/line of argument analytic focus allowed for comparison and synthesis across studies.

While the review was not prospectively registered, transparency was supported through an a prior project analysis guide and staged analytic procedures, reported in this section and in the **Supplementary Materials**.

### 2.2 Eligibility criteria

We defined eligibility criteria to prioritise studies of qualitative relevance and conceptual contribution to therapeutic mechanisms. Included studies were required to report primary qualitative data using personal accounts to examine relationships with companion animals in relation to mental health experiences. Participants were required to have a diagnosed mental health condition or to report significant emotional distress, including those with co-morbid mental health difficulties associated with a primary physical long-term condition. Mental health was defined broadly and included diagnosed and undiagnosed mental health conditions, severe psychological distress (i.e., self-harm and suicidality), and mental health experiences in the context of long-term physical illness. Studies were restricted to those published in English and up to April 2025.

Studies were excluded if they were reviews, opinion pieces, books or book chapters, purely quantitative, or if they examined animal-assisted interventions without participant ownership of the animal. Full inclusion and exclusion criteria are provided in **Table 1**.

**Table 1:** Inclusion and exclusion criteria.

Inclusion criteria	Exclusion criteria
English language paper.	Not an English language paper.
Primary data.	Not primary data (e.g., systematic or review article/opinion piece).
Peer-reviewed journal article/conference paper/research dissertation.	Not a peer-reviewed journal article (e.g., books/book chapters).
Relate to pet ownership and domestic animals.	Studies unrelated to pet ownership (e.g., animal-assisted therapy, which does not involve the direct ownership of domestic animals).
Related to the impact of pet ownership on mental health conditions or co-morbid mental health related to long-term physical conditions.	Not related to the impact of pet ownership on mental health conditions or mental health components of long-term physical conditions, or the nature of the sample was unclear.
Qualitative methods only or qualitative elements of studies relating to pet owners.	Quantitative methods or mixed methods studies.

### 2.3 Search strategy and information sources

The review incorporated qualitative studies from a prior systematic review (8), which searched nine electronic databases (ASSIA, CINAHL Plus, Embase, International Bibliography of the Social Sciences, Medline, PsycINFO, Social Science Full Text, Sociological Abstracts, and Web of Science) from the earliest record to March 2017. To identify studies published since the original review, updated searches were conducted in April 2025 using Google Scholar, which provides broad coverage of interdisciplinary research and grey literature across psychiatry, psychology, sociology, public health, and human-animal studies (21). Given the narrative and interpretive aims of the review, the updated search was intended to maximise conceptual relevance and disciplinary breadth rather than reproduce the exhaustive database architecture of the original systematic review. The search was therefore used to identify qualitative studies capable of contributing to the development of the mechanisms-focused synthesis.

Updated searches covered publications from 2017 to 2025. The April 2025 update used the same core pet ownership and mental health search concepts as the original systematic review, with an additional qualitative research block to focus retrieval on studies reporting qualitative data. Search terms were organised into three areas: pet ownership, diagnosed mental health conditions or co-morbid mental health difficulties associated with long-term physical conditions, and qualitative research methods. Terms were used iteratively and in combination in Google Scholar, reflecting the dispersed and interdisciplinary nature of the literature. Full search terms, including phrase searching and truncation, are provided in **Supplementary Material 1**. Long-term physical condition terms were included because studies were eligible where mental health difficulties were examined in the context of co-morbid or condition-related psychological distress.

### 2.4 Study selection

**Figure 1:**
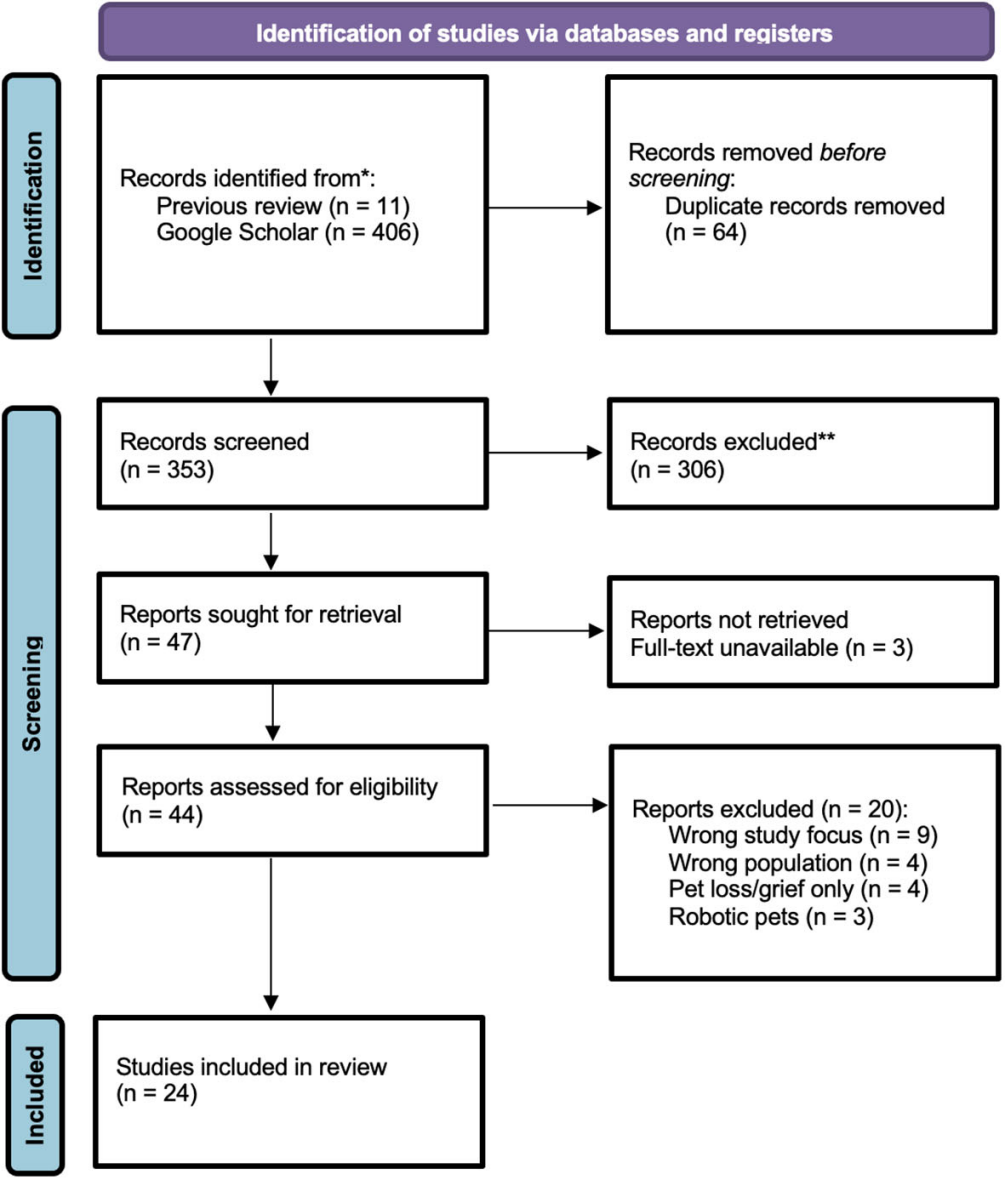
PRISMA diagram showing study selection process.

Search results were uploaded to Microsoft Excel and duplicates removed. Title and abstract screening was conducted independently by the first and third authors, resulting in the assessment of 353 records. Following screening, 44 articles were retained for full-text review and were randomly distributed across the author team (Author 1 = 15; Author 2 = 14; Author 3 = 15). Each author read the assigned papers in full and made inclusion/exclusion decisions based on eligibility criteria. Across the screening process, uncertainties or disagreements were resolved through discussion with the full author team until consensus was reached. Full-text assessment resulted in the exclusion of 20 articles, leaving 24 qualitative studies included in the final synthesis (see PRISMA diagram in **Figure 1**). Study characteristics are summarised in **Table 2**.

**Table 2:** Characteristics of included studies (n = 24).

Author (year)	Country	Design and analysis	Participants (n)	Type of pet	Mental health condition (context)
Brooks et al. (2019) (33)	UK	Descriptive qualitative Critical discourse analysis (CDA)	12	Dogs; Cats; Birds; Hamsters; Guinea pigs	SMI^1^
Schmitz et al. (2022) (25)	USA	Phenomenological Thematic analysis	17	Companion animal*	Severe psychological distress^2^
Young et al. (2020) (28)	Australia	Participatory/empowerment descriptive Thematic analysis drawing on grounded theory	35	Dogs; Cats; Birds; Reptiles	Severe psychological distress^2^; trauma-related distress^3^
Scanlon et al. (2021) (44)	UK	Semi-structured interviews; thematic analysis	20	Dogs	SMI1; severe psychological distress^2^ (substance misuse)
Fossey et al. (2020) (45)	Australia	Narrative inquiry, thematic analysis	29	Dogs; Cats	SMI1 (homelessness)
Kosteniuk & Dell (2020) (32)	Canada	Semi-structured interviews, thematic analysis	7	Dogs; Cats	Trauma-related distress^3^; anxiety; depression (substance use disorder)
Love (2021) (27)	USA	Inductive thematic analysis	149	Dogs; Cats; Rabbits; Guinea pigs	Severe psychological distress^2^; trauma-related distress^3^; anxiety; depression
Brooks et al. (2016) (7)	UK	Framework analysis informed by Corbin & Strauss’s Illness Work and personal communities approach	54 interviewed; 25 pet owners included in pet-specific analysis	Dogs; Cats; Birds; Hamsters; Guinea pigs; Rabbits	SMI^1^
Gaughan (2021) (46)	USA	Qualitative – phenomenological approach to analysis. Provisional coding approach	8	Dogs (ESA)	Anxiety
Williamson et al. (2022) (47)	Canada	Exploratory patient-oriented, longitudinal time series research design. Qual – semi-structured interviews and deductive content analysis.	5	Dogs (service dogs)	Trauma-related distress^3^ (substance use disorder)
Hawkins et al. (2024) (16)	UK	Qualitative – semi-structured design. Reflexive thematic analysis.	16	Dogs; Cats	Anxiety and depression
Barcelos et al. (2021) (48)	UK	Qualitative analysis, interviews and thematic analysis.	36	Dogs	Neurodivergence^4^; severe psychological distress^2^
Wisdom et al. (2009) (24)	USA	Mixed methods. Interviews analysed using grounded theory	101 pet owners	Dogs; Cats; Birds; Rabbits; Horses; Guinea pigs	SMI^1^
Zimolag (2017) (49)	USA	Qualitative; descriptive exploratory design informed by occupational therapy perspectives; interviews analysed thematically	10	Dogs; Cats	SMI^1^
Schmitz et al. (2021) (26)	USA	Qualitative; semi-structured interview narratives analysed using a minority resilience framework	45	Dogs; Cats; Birds; Reptiles; Rodents	Anxiety; depression; trauma-related distress^3^ (LGBTQ+ minority stress)
von Humboldt et al. (2024) (36)	Portugal	Qualitative study using semi-structured interviews analysed via qualitative content analysis	351	Dogs; Cats; Birds	Anxiety; depression (COVID-19)
Zablan et al. (2023) (50)	USA	Qualitative; descriptive design using semi-structured interviews analysed via reflexive thematic analysis	22	Dogs; Cats	Anxiety; depression (older adults, COVID-19)
Gan et al. (2020) (51)	UK	Qualitative; exploratory design using semi-structured interviews analysed via thematic analysis	19	Dogs; Cats	Anxiety; depression (older adults)
Cyr & Hawkins (2024) (29)	Canada	Qualitative; descriptive design using semi-structured interviews analysed via reflexive thematic analysis	23	Dogs; Cats	Anxiety; depression (perinatal)
Garland-Lewis et al. (2024) (30)	USA	Qualitative; participatory photovoice design with group discussions and reflexive thematic analysis	17	Dogs	Anxiety; depression; trauma-related distress^3^ (homelessness)
Hawkins et al. (2021) (31)	UK	Qualitative; semi-structured interviews analysed using reflexive thematic analysis	54	Companion animal*	Severe psychological distress^2^; anxiety; depression
Kerr-Little et al. (2023) (52)	UK	Qualitative; semi-structured interviews analysed using reflexive thematic analysis	22	Dogs	Chronic mental illness^5^ (substance use disorder)
Kabel et al. (2015) (35)	USA	Qualitative; narrative interview design analysed using thematic analysis	18	Dogs; Cats	Anxiety; depression (HIV)
Sudbury-Riley (2024) (34)	UK	Qualitative; in-depth interviews analysed using reflexive thematic analysis	40	Companion animal*	Anxiety; depression (COVID-19)

*Not otherwise specified.

^1^ SMI, Severe Mental Illness (studies centred on conditions typically characterised by psychosis, bipolar disorder, or schizophrenia-spectrum presentations, involving marked functional impairment/engagement with secondary mental health services).

^2^ Severe psychological distress = studies foregrounding acute or persistent distress characterised by suicidality, self-harm, or overwhelming emotional suffering, irrespective of formal diagnosis, where survival rather than recovery was the dominant concern.

^3^ Trauma-related distress = studies focused on psychological distress associated with exposure to traumatic events, including post-traumatic stress disorder and complex trauma presentations.

^4^ Neurodivergence = studies in which participants were primarily described in relation to neurodevelopmental conditions (ASD, ADHD), with mental health needs understood in the context of neurodivergent functioning rather than episodic psychiatric disorder.

^5^ Chronic mental illness = studies emphasising long-term, enduring mental health difficulties that were not defined primarily by psychosis or acute crisis, in which chronicity, persistence, or cumulative impact over time was a central analytic feature.

### 2.5 Data extraction

Data extraction was conducted collaboratively by all authors using a structured spreadsheet developed for this review. Extracted data included study characteristics, participant populations, mental health context, companion animal type, qualitative methodology, authors’ analytic interpretations, and verbatim participant quotations. Extraction prioritised verbatim quotations as the principal analytic material underpinning mechanism development within the synthesis.

Following initial extraction, the first author reviewed and verified all extracted data across studies and compiled the final consolidated extraction spreadsheet used for analysis.

### 2.6 Evaluation of qualitative studies

Included qualitative studies were evaluated by AR and HB using criteria developed by Blaxter (1996) for the appraisal of qualitative research (22). This approach is well established within applied social science and health services research and was considered appropriate for the interpretive orientation of the review, where the analytic focus was on meaning, context, and the contribution of participant accounts to mechanism development.

Evaluation focused on whether studies provided an adequate description of context and participants, transparent analytic procedures, evidence to support findings through quotations, observations, or exemplars from the data, and steps to support credibility and consistency of interpretation. These criteria were used to assess the contribution and interpretive adequacy of studies rather than to impose exclusionary quality thresholds.

The CASP qualitative checklist was not used as the primary appraisal framework because its checklist-driven format was considered less suited to the interpretive aims of this narrative synthesis. This decision is consistent with critiques cautioning against overly procedural uses of CASP in qualitative evidence synthesis, particularly where appraisal needs to attend to meaning, context, and analytic contribution rather than methodological compliance alone.

### 2.7 Analytic synthesis

Analytic synthesis followed a framework-based narrative approach, with a line-of-argument synthesis implemented through a staged, iterative process. The evaluation of qualitative studies informed the interpretation and weighting of evidence during synthesis, particularly where studies varied in contextual detail, transparency of analysis, and the extent to which analytic claims were supported by participant quotations or other data exemplars. The analytic strategy was specified in advance in the project analysis guide (see **Supplementary Material 2**), with staged procedures used to support transparency in movement from extracted data to higher-order synthesis (20, 23). The first author led the analytic synthesis, including the organisation of extracted data, development of mechanism statements, clustering of mechanisms, and drafting of the line-of-argument synthesis. AR and HB contributed to the evaluation of included studies, interpretation of emerging mechanism clusters, refinement of the framework, and validation of the developing synthesis through iterative discussion.

#### 2.7.1 Developing the framework

Following full-text reading of all included studies, and prior to formal data extraction, the research team undertook preliminary analytic work to support the subsequent synthesis. During this phase, the findings and discussion sections of included papers were examined to identify how companion animal support was being implicitly or explicitly framed in relation to therapeutic and psychosocial processes, such as emotional regulation, behavioural activation, relational safety, responsibility, and meaning-making. On the basis of this familiarisation, the team developed a bespoke table of therapy types, definitions, and mechanisms, collating commonly referenced literature on descriptions of psychological and psychosocial intervention approaches alongside their proposed core mechanisms of action. This table was developed to support an analytically detailed comparison of specific aspects of how therapeutic interventions work rather than to imply that companion animals’ support is equivalent to formal therapy.

This framework (see **Table 3**) was generated inductively from the included studies and used as an analytic reference tool rather than a coding frame. Its purpose was to sensitise subsequent extraction and synthesis to points of convergence, partial overlap, and divergence between companion animal mechanisms and formal therapeutic mechanisms, while retaining analytic openness to mechanisms that did not map neatly onto existing therapeutic models.

**Table 3:** Analytic framework of therapeutic approaches and core mechanisms used to sensitise synthesis.

Therapy type	Definition	Core mechanism(s)	How it might look from pets	Comparison
Cognitive Behavioural Therapy (CBT)	A structured, goal-oriented psychotherapy that aims to identify and modify unhelpful thoughts and behaviours.	Cognitive restructuring, behavioural experiments, exposure, behavioural activation.	Pets provide opportunities to challenge negative thinking (e.g., “I’m a burden” vs “my pet depends on me”), reduce avoidance (e.g., leaving the house to walk a dog), and reinforce helpful routines.	CBT combines both cognitive and behavioural strategies. It is broader than Behavioural Activation, which focuses solely on the behavioural component.
Behavioural Activation (BA)	A subset of CBT that focuses on increasing engagement in positively reinforcing activities to counteract depression.	Activity scheduling, mood tracking, exposure to rewarding tasks.	Feeding, walking, or caring for a pet introduces purposeful daily activities even during low mod.	Unlike full CBT, BA does not directly target thoughts. It assumes that improved mood can follow from changes in behaviour alone.
Dialectical Behavioural Therapy (DBT)	A therapy developed for individuals with high emotional sensitivity and dysregulation. Blends CBT techniques with mindfulness and validation.	Emotional regulation, distress tolerance, interpersonal effectiveness, mindfulness.	Pets can help distract from crisis urges, offer comfort during emotional surges, or teach consistency and validation.	Differs from CBT by integrating acceptance strategies and focusing more on emotion regulation than cognitive restructuring.
Schema Therapy	An integrative model for addressing entrenched maladaptive patterns (“schemas”) rooted in early life experiences.	Identifying schemas, limited reparenting, experiential exercises, mode work.	Pets may offer corrective emotional experiences, countering schemes of abandonment or defectiveness.	Incorporates CBT, attachment theory, and psychodynamic principles, but with more focus on early unmet needs than CBT or DBT.
Mindfulness-Based Therapies	Therapies that promote awareness of the present moment with a non-judgmental stance (e.g., MBSR, MBCT)	Attentional control, acceptance, emotion regulation.	Being with a pet can draw attention to the present (stroking fur, hearing breathing), helping to regulate overwhelming thoughts.	Differs from CBT by not challenging thoughts, but accepting them. Often integrated into other models like DBT or ACT.
Compassion-Focused Therapy (CFT)	Designed for people with high shame and self-criticism, this approach helps develop a compassionate inner voice.	Soothing system activation, self-kindness, compassion imagery.	Pets model acceptance and non-judgment, helping people practice warmth toward themselves.	Differs from CBT and mindfulness therapies by explicitly targeting shame and using evolutionary psychology.
Psychoanalytic/Psychodynamic Therapy	Focuses on unconscious patterns and relational dynamics that stem from early life experiences	Insight, transference, emotional expression, relational patterns.	Pets may elicit early attachment dynamics or help individuals explore unmet needs in a safe context.	Slower-paced and insight-focused, contrasting with more action-oriented models like CBT or DBT.
Emotion-Focused Therapy (EFT)	Centres on deepening emotional awareness and processing to support change and relational healing.	Emotion identification, expression, transformation.	Pets offer a safe outlet for emotion and can help break patterns of emotional suppression.	EFT differs from CBT by focusing on the experience and regulation of emotion, rather than changing thought patterns.
Attachment-Based Therapy	Aims to repair disrupted attachment patterns and develop secure, trusting relationships.	Emotional security, trust, consistency, relational repair.	Pets may act as reliable, emotionally safe figures, particularly for those with anxious or avoidant attachment styles.	Overlaps with psychodynamic therapy in its focus on relational history but is more explicitly grounded in attachment theory.
Narrative Therapy	Helps clients re-author their identities and stories, separating them from problem-saturated narratives.	Externalisation, identity development, meaning-making.	Pets may be narrated as “lifesavers” or anchors in recovery, helping shift a person’s identity towards survival, caregiving, or strength.	Unlike CBT and psychodynamic therapy, it avoids pathologizing language and focuses on language and story rather than symptoms or inner drives.
Solution-Focused Therapy	Brief, future-oriented therapy that builds on client strengths and identifies what works.	Exception-finding, scaling, goal-setting.	Pets may highlight daily successes (e.g., “I got out of bed to feed them”), reinforcing small wins and future-oriented progress.	Differs from problem-focused therapies like CBT; avoids dwelling on causes or pathology.
Existential Therapy	Explores human concerns such as meaning, mortality, freedom, and isolation.	Authenticity, personal responsibility, finding meaning.	Pets can give life meaning, reduce existential loneliness, and remind people of interdependence and presence.	Unlike CBT or psychodynamic models, existential therapy is philosophical rather than symptom-based.
Humanistic Therapy	Centres on personal growth, self-actualisation, and the inherent worth of each person.	Unconditional positive regard, empathy, authenticity.	Pets offer unconditional acceptance, foster self-worth through relational presence.	Shares person-centred values with CFT but less structured and not explicitly shame-focused.
Interpersonal Therapy (IPT)	Time-limited therapy focused on improving communication and addressing social role issues.	Grief, role transitions, interpersonal conflict, social skill-building.	Pets help buffer isolation, ease transitions (e.g., bereavement), and reinforce a sense of companionship.	More relational than CBT but more structured and short-term than psychodynamic therapy.
Trauma-Informed Therapy	Not a model in itself, but a framework that prioritises safety, empowerment, and trauma awareness across therapeutic contexts.	Safety, trustworthiness, choice, collaboration.	Pets offer consistent, safe relationships that can support regulation and healing from traumatic experiences.	Often layered into other modalities (e.g., CBT, EMDR, psychodynamic), but ensures all work avoids re-traumatisation.
Recovery-Oriented/Strengths-Based Therapy	An approach rooted in mental health recovery movements that emphasises identity, hope, and purpose beyond symptom reduction.	Empowerment, strengths, identity reconstruction, autonomy.	Pets provide a role (carer, protector), a reason to keep going, and pride in managing responsibility.	Differs from traditional clinical models by resisting deficit-based frameworks.
Family Therapy	Systemic therapy that views the family unit – not the individual – as the locus of change.	Patterns, roles, communication, systemic feedback.	Pets can function as emotional bridges, redirect attention during conflict, or redistribute care roles.	Unlike individual therapies, family therapy focuses on relational dynamics and shared narratives.
Sensory-based/Tactile Therapies	Use sensory input (touch, temperature, texture) to support regulation, especially in trauma or neurodivergence.	Grounding, sensory integration, embodied presence.	Petting or lying near an animal can reduce arousal, support co-regulation, and bring awareness to bodily sensations.	Focuses on body-based regulation rather than cognition (CBT) or talk-based insight (psychodynamic).
Occupational Therapy/ADL-Focused Work	Supports re-engagement with meaningful daily activities and life roles.	Routine, role fulfilment, independence, identity through action.	Pet care (feeding, walks, appointments) provides a scaffold for daily structure and function.	Often complements psychological therapy by focusing on doing rather than insight or emotion.
Motivational Interviewing (MI)	A collaborate approach designed to strengthen a person’s motivation for change.	Ambivalence resolution, values clarification, change talk.	Pets may anchor a person’s reasons for change (e.g., sobriety to continue care), helping resolve ambivalence.	MI is non-confrontational and directive, unlike CBT which assumes readiness to change.
Grief Therapy/Bereavement Support	Supports individuals in processing and integrating loss, especially when grief is complex or prolonged.	Meaning-making, continuing bonds, emotional expression.	Pets provide support during grief but also become the focus of grief; mourning pets can trigger earlier unprocessed losses.	Distinct from general talk therapy through its focus on loss-specific processes.
Group Therapy	A format (not a model) where therapeutic mechanisms arise through peer interaction and shared experience.	Universality, belonging, feedback, social learning.	Pets can serve as shared points of conversation, emotional grounding, or metaphors in animal-assisted group work.	Delivery format rather than theoretical model; can be used with most modalities (CBT group, psychodynamic group, etc.)
Animal-Assisted Therapy (AAT)	Structured therapeutic work involving trained animals, delivered by a qualified therapist with a goal-directed plan.	Engagement, emotional safety, non-verbal connection.	While not formal AAT, home pets may mimic some AAT functions – emotional regulation, safety, motivation – informally.	Different from companion pet support in being structured, formalised, and goal-specific.

#### 2.7.2 Framework-based synthesis

Formal synthesis addressed how people living with mental health difficulties describe companion animals as supporting emotional distress, daily functioning, and sense of self; what perceived therapeutic mechanisms can be identified across these qualitative accounts; and what these accounts suggest about forms of support that may be difficult to provide through time-limited, verbally mediated, professionally delivered interventions.

The synthesis comprised three interconnected phases, moving from descriptive categorisation to theoretical mapping, and then to critical interpretation and line of argument.

##### 2.7.2.1 Phase 1: mechanism identification and clustering

Mechanism statements were extracted from each included study and entered into a coding spreadsheet, with one discrete mechanism per row. Through iterative reading and comparison, mechanisms were grouped into provisional clusters based on conceptual similarity. Clustering was informed by the data rather than imposed *a priori*, though attention was also paid to constructs commonly identified in the human-animal bond literature, including presence and companionship, emotional regulation, routine and behavioural structure, responsibility and obligation, identity and role, meaning and purpose, safety and trust, social mediation, and sensory or tactile processes. Each cluster was defined, and illustrative quotations were identified from the supporting data. The distribution of clusters across studies and populations was noted to examine patterns of convergence and specificity.

##### 2.7.2.2 Phase 2: mapping onto formal therapeutic mechanisms

A mapping matrix was constructed to examine relationships between the identified mechanism clusters and mechanisms targeted by formal psychological therapies. Therapies were selected for inclusion in the mapping based on their representation in the extracted data; these included behavioural activation, attachment-based therapy, compassion-focused therapy, dialectical behaviour therapy (specifically distress tolerance), existential therapy, humanistic and person-centred approaches, mindfulness-based approaches, and trauma-informed frameworks. For each cluster-therapy pairing, the nature of the relationship was examined: whether the companion animal relationship approximated the therapeutic mechanism, instantiated it through different means, or bore only a superficial resemblance. This phase drew on a therapy mapping framework further and articulated the core mechanisms of each therapy modality and how these might manifest in pet relationships.

##### 2.7.2.3 Phase 3: identifying mechanisms beyond formal therapy

The final analytical phase examined how companion animal relationships appeared to provide therapeutic mechanisms that were unavailable, constrained, limited or diluted within formal therapeutic relationships. Data were re-examined for explicit contrasts between pet and human or professional relationships, for mechanisms with no clear therapeutic equivalent, and for those in which therapeutic approximation was limited by professional boundaries, time constraints, or the verbal and reflective demands of formal intervention. Particular attention was paid to evidence concerning survival and continuity—how companion animals sustained the everyday living in the absence of hope, insight, or recovery-oriented progress—as this material carried distinct theoretical significance.

##### 2.7.2.4 Synthesis

Findings from the three phases were synthesised into a narrative structured around the argument that companion animals enact mechanisms recognisable from formal therapy, yet also provide forms of support that challenge assumptions about what constitutes effective mental health intervention. The synthesis, presented in Section 3, prioritised transparency by retaining coding materials, grounding all claims in specific studies and quotations, and acknowledging areas where evidence was limited.

## 3 Results

The results are presented as a narrative synthesis organised around analytically derived mechanisms. Section 3.1 provides an analytic overview of the mechanism clusters and their evidential coverage, followed by a systematic examination of mechanisms ordered by analytic centrality and functional dependence. Throughout, attention is paid to the co-occurrence of mechanisms, variation across mental health contexts, and the distinction between mechanisms that sustain day-to-day functioning and those that operate under conditions of severe psychological distress and survival threat.

### 3.1 Analytic overview of mechanism clusters

To orient interpretation of the results, **Table 4** summarises the mechanism clusters identified across the included qualitative studies, indicating their conceptual focus, evidential coverage (unique contributing studies; n = 24), and the primary mental health contexts represented. Definitions of mental health context categories are provided in the table note to clarify analytic boundaries where terminology may be ambiguous. To further support transparency, **Supplementary Material 3** provides a study-by-mechanism contribution matrix indicating which included studies contributed evidence to each mechanism cluster.

**Table 4:** Perceived mechanism clusters through which companion animals supported mental health across included qualitative studies (n = 24).

Mechanism number and cluster	Analytic definition	Relevant studies (n)	Primary mental health contexts presented	Illustrative analytic indicators
1. Emotional regulation	Down-regulation or containment of distress through comfort, distraction, soothing, or affective co-regulation	23	Depression; Anxiety; SMI; Trauma-related distress; Severe psychological distress	Calming; distraction from distress; affective containment
2. Routine and structure	Temporal organisation of daily life through non-optional care practices that scaffold functioning	23	Depression; Anxiety; SMI; Chronic mental illness; Severe psychological distress3	Feeding; walking routines; daily structure; enforced activity
3. Safety and trust	Experience non-judgment, predictability, reliability, and emotional safety, enabling vulnerability and stability	22	Trauma-related distress; Anxiety; SMI; Severe psychological distress	Unconditional acceptance; reliability; reduced fear of evaluation
4. Meaning and reason to live	Existential anchoring that renders life tolerable or morally necessary, particularly under crisis conditions	16	Severe psychological distress; SMI; Chronic mental illness	Purpose; “reason to live”; survival anchoring
5. Identity and role	Reconstruction of self through caregiving identities and valued roles that counter stigma or diminished self-worth	13	Chronic mental illness; SMI1; Depression	Carer and protector identity; competence; feeling useful
6. Practical support and access	Material or functional support that increases participation, mobility, or access to environments and services	5	Chronic mental illness; SMI	Leaving the house; functional scaffolding
7. Responsibility and obligation	Moral duty to another being that compels action even when self-directed motivation is absent	5	Severe psychological distress; SMI; Depression	“They depend on me”; non-negotiable duty
8. Social mediation	Facilitation of social contact or stigma buffering through animal-mediated interaction	5	Anxiety; Chronic mental illness; SMI	Social bridge; legitimising presence
9. Presence and companionship	Relational co-presence framed as companionship or “being with,” independent of explicit regulation or safety claims	4	Depression; Anxiety; Chronic mental illness	Not alone; constant companionship
10. Sensory/tactile	Embodied regulation primarily via touch, warmth, and somatic grounding (distinct from broader emotional regulation)	1	Trauma-related distress; Neurodivergence	Stroking; warmth; bodily grounding

Study counts refer to the number of unique qualitative studies contributing evidence to each mechanism cluster. Mechanisms frequently co-occurred within individual accounts; clusters are analytically distinct but relationally interdependent.

As summarised in **Table 2**, several mechanisms - notably emotional regulation, routine and structure, safety and trust - were evident across the majority of included studies and spanned differing mental health contexts, indicating their central role in how companion animals support mental health. Other mechanisms, including identity and role reconstruction or reasons to live, were represented in fewer studies but carried disproportionate analytic significance, particularly within the contexts of chronic mental illness and severe psychological distress. Mechanisms were rarely described as discrete or sequential; participants’ accounts instead depicted interlocking processes through which presence, regulation, obligation, and meaning mutually reinforced one another. To reflect these patterns, the sections that follow are organised according to analytic centrality and functional dependence, beginning with mechanisms that operate as relational preconditions for other forms of support before turning to those that emerge under conditions of heightened vulnerability and crisis.

### 3.2 Core mechanisms: relational and regulatory foundations

The mechanisms described in this section represent the foundational analytic core of the synthesis. Evident across the majority of included studies (n = 24), these mechanisms operate as relational preconditions that render other forms of support possible. By establishing conditions of emotional containment, safety, and trust, these mechanisms create the relational infrastructure through which subsequent mechanisms—routine, responsibility, and meaning—can emerge and be sustained.

#### 3.2.1 Emotional regulation as relational co-regulation

Emotional regulation emerged as the most frequently reported mechanism, evident in accounts spanning depression, anxiety, severe mental illness, trauma-related distress, and severe psychological distress. Regulation was rarely framed as an internally generated skill or cognitively mediated process. Instead, participants described emotional regulation as arising through relational co-presence with their animals, positioning animals as active contributors to affective containment rather than passive sources of comfort.

Accounts emphasised calming, soothing, and distraction from distress, particularly during periods of heightened anxiety or crisis. These regulatory effects were not described as the outcome of a deliberate strategy. Rather, regulation was described as occurring with the animal, through proximity, shared space, and embodied interaction:

“When I was depressed she would lay down on the bed next to me and put her paw across me, and I didn’t even have to tell her I was depressed, she’d just sense it.”

*(Wisdom* et al.*, 2009, p. 6) (*24)

Regulation was frequently enacted through embodied and sensory means, including physical closeness, rhythmic interaction, and attentiveness to the animal’s presence. Participants described being calmed by the presence, touch, or attunement to the animal’s movements, particularly when verbal expression was perceived as effortful or unavailable:

“I feel like they have this chemical that they can sense. Like if you’re having a bad day or something and they just want to come to you, they just want to help [ … ] sometimes if I’m crying, having a bad day, she’ll come on the couch and just cuddle with me.”

*(Schmitz* et al.*, 2022, p. 703) (*25)

This emotional regulation did not necessitate a future-oriented stance or hope for recovery. Regulation was immediate and situational, enabling distress to be endured rather than resolved. In studies involving severe psychological distress, this capacity to contain emotion—rather than transform it—was especially prominent.

Emotional regulation was closely linked with safety and trust. Participants’ willingness to remain physically and emotionally close to their animals during distress was underpinned by expectations of non-judgement and reliability. Perceived safety within the relationship enables co-regulation, serving as a relational condition for engaging with other forms of support.

#### 3.2.2 Safety and trust as preconditions for support

Safety and trust emerged as mechanisms underpinning participants’ capacity to engage with other forms of support. Animals were described as non-judgmental, predictable, and emotionally safe, enabling forms of closeness absent from many human relationships. Safety was not described as something that developed gradually; rather, it was treated as an assumed property of the relationship itself.

Participants frequently contrasted animal with human relationships, with the latter viewed as characterised by evaluation or the risk of negative judgment. Animals offered acceptance without scrutiny:

“Animals are so much better at empathising because they’re not worried about their own stuff [ … ] they’re just like ‘well, you’re my person and I care about you.’”

*(Schmitz* et al.*, 2021, p. 3070) (*26)

This emotional reassurance made vulnerability possible, enabling participants to remain with their animals when feeling emotionally overwhelmed or in crisis without needing to justify or manage their emotional state.

Trust was linked to predictability and reliability. Animals were described as always present and emotionally responsive, with responses that did not fluctuate with a person’s mental state or behaviour. This predictability was particularly relevant during psychological distress, where participants described heightened sensitivity to relational threat. At these times, animals were positioned as emotionally safe companions because they did not withdraw, retaliate, or demand explanation. In several accounts, this sense of safety was described as life-sustaining:

“[Companion animals] felt like my only friends at times and were a huge part of my life. Sometimes they would be the only reason I wouldn’t attempt suicide.”

*(Love, 2021, p. 179) (*27)

These accounts highlight safety and trust as needed for continued survival—a positioning that differentiates animal relationships from many therapeutic models, where safety is framed as something to be established through alliance-building over time.

### 3.3 Structuring daily life and agency

Building on the pre-conditions of safety, trust, and relational co-regulation, a second set of mechanisms emerged through participants’ everyday interactions with their animals. These operated at the level of action and agency, shaping how participants organised their daily lives in the face of ongoing distress. These forms of action were not experienced as deliberate self-management strategies. Instead, they arose through felt obligation, compelling participants to act in ways that sustained routine and responsibility even during periods of profound psychological difficulty.

#### 3.3.1 Routine and structure as non-optional temporal scaffolding

Routine and structure sustained engagement with daily life. Participants described how caring for companion animals imposed regular temporal demands which structured an otherwise formless daily life. These routines were not framed as self-initiated coping strategies but experienced as inbuilt obligations anchored in the needs of another being, persisting even when motivation, energy, or hope were depleted.

Participants described daily practices such as feeding, walking, and attending to animals’ needs as the primary forces organising their time:

“If it wasn’t for Elvis [dog] wanting to get up and walk every morning, I would probably just sit home here in the four walls.”

*(Young* et al.*, 2020, p. 196) (*28)

Here, routine does not function as behavioural activation in the conventional sense. Action was not initiated by anticipated benefit or therapeutic rationale, but by the animal’s ongoing and unavoidable needs. Routine operated as a temporal organisation constraining withdrawal without requiring planning or internal motivation. Care tasks continued even when participants felt personally hopeless:

“Needing to walk the dog got me out of bed even on the darkest days [ … ] walking her ensured I got fresh air and exercise, both vital to my wellness.”

*(Cyr & Hawkins, 2024, p. 751) (*29)

This temporal scaffolding endures despite fluctuations in mood, cognition, or self-care capacity. The animal’s dependence rendered routine morally compelling rather than optional.

#### 3.3.2 Responsibility and obligation as relationally located agency

Responsibility and obligation emerged as relationally compelled action, acquiring moral weight and durability. Responsibility-oriented action toward another being’s ongoing needs generated a sense of obligation that participants described as impossible to ignore. Animals were dependent on participants in ways that could not be deferred or delegated, even during severe psychological distress. Care was not framed as reciprocal exchange or chosen role adoption, but as a binding commitment that persisted regardless of internal states:

“I will eat ramen for the rest of my life before I betray my promise to give them a forever home.”

*(Garland-Lewis* et al.*, 2024, p. 11) (*30)

Responsibility served as a moral anchor, compelling engagement with life and overriding impulses toward withdrawal, disengagement, or self-harm. In this sense, responsibility operated as an externally located agency, sustaining action when self-directed motivation was absent. In several studies, responsibility was explicitly linked to survival:

“They give me a reason to live each day. Without them I would probably consider taking my own life.”

*(Hawkins* et al.*, 2021, p. 552) (*31)

Here, obligation constrains action in the present, anchoring participants to continued existence through the immediate ethical demand of care. Responsibility functioned as a stabilising mechanism rather than a transformative one, sustaining engagement with life without requiring belief in improvement. This responsibility was not viewed as interchangeable with other forms of obligation (e.g. employment or family roles). Responsibility toward animals was described as unconditional, durable, and morally non-negotiable. Sustained care practices contributed to participants’ sense of themselves as needed and reliable, laying the groundwork for identity and meaning-making processes.

### 3.4 Identity, role and meaning

The relational security and sustained action described above fed back into participants’ sense of self, role, and existential orientation. Sustained engagement in caring relationships reshaped how participants understood themselves—not through reflection or therapeutic insight, but through ongoing enactment of care, responsibility, and presence.

Identity and meaning arose from what participants did for their animals and who they became in relation to them over time. Companion animal relationships supported mental health not by stabilising emotion or organising daily life, but by enabling participants to inhabit viable forms of selfhood under conditions in which identity had otherwise been eroded.

#### 3.4.1 Identity and role reconstruction

Identity and role reconstruction translated into longer-term changes in participants’ sense of self. Participants described how ongoing responsibility for a companion animal enabled them to occupy socially and morally legitimised roles— carer, protector, or provider—under conditions in which other identity positions had been lost or destabilised.

Participants described becoming ‘someone who looks after’ another being, a role conferring a sense of competence, reliability, and worth that contrasted with experiences of dependency, failure, or stigma in other domains of life:

“[Companion animals] make me feel like regardless of all the other things I do wrong, at least in this one creature’s eyes, I’m doing the best I can.”

*(Schmitz* et al.*, 2022, p. 704) (*25)

These caregiving identities were forged and reinforced daily through feeding, walking, protection, and decision-making. This practical grounding appeared central to their durability: participants did not need to hold a stable belief in their own value; value was articulated through care. In this way, identity reconstruction functioned as a property of relational obligation not as an internally generated cognitive shift.

Participants also described these roles as morally weighted. Being responsible for an animal positioned individuals as trustworthy, dependable, and needed—qualities frequently experienced as absent in relationships with institutions, services, or family members:

“My best quality is that I love animals and I take care of animals.”

*(Wisdom* et al.*, 2009, p. 7) (*24)

Identity reconstruction was linked to mechanisms of routine and responsibility described in Section 3.3. This organisation of daily life created repeated opportunities to provide care, while the non-negotiable nature of responsibility sustained this during periods of low motivation or distress. Over time, such practices consolidated into stable identity positions that participants described as protective against further erosion of self.

#### 3.4.2 Meaning, purpose and reasons to live

Meaning and purpose emerged as mechanisms through which relational practices and role-based identities acquired existential significance. Participants described companion animals as orienting their lives around something worth sustaining, even when distress was persistent, recovery felt implausible, or human relationships were experienced as unreliable. Meaning was not framed as cognitive reframing or through a narrative of ‘growth,’ but as practically grounded and relationally anchored, arising from ongoing responsibility, attachment, and the moral consequences of continued presence.

Participants located purpose in the everyday of ‘being needed’. Identity reconstruction centred on occupying valued roles, meaning-making extended this by positioning roles as reasons to continue and remain engaged with life. Companion animals were described as providing an organising centre that reconfigured what life was ‘for’:

“They give me a reason to live, for them, to live for myself, and to live for my family, and you know, change.”

*(Kosteniuk & Dell, 2020, p. 97) (*32)

A subset of accounts framed meaning as a constraint on self-harm and suicide. In these narratives, companion animals were described as placing moral limits on withdrawal, disappearance, or death because of the anticipated consequences for a dependent other. Meaning-making, therefore, operated as a positive orientation toward life and a prohibition against suicide:

“There have been several times where I’ve had severe suicidal ideations, and I see my cats and be like, ‘okay, I can’t die yet because I got to take care of my cats first.’”

*(Schmitz* et al.*, 2022, p. 706) (*25)

Meaning and purpose were temporally stabilising, creating continuity through time—something to return to daily and something to remain for—counteracting foreshortened futures associated with severe distress. Meaning did not necessarily entail hope but reasons to remain, grounded in relational obligation rather than anticipated recovery.

### 3.5 Survival and crisis as a distinct analytic register

Participants’ accounts of survival during periods of acute psychological risk warrant analytic treatment in their own right. Participants described companion animals as playing a decisive role during moments characterised by suicidality, intense self-harm urges, dissociation, or emotional collapse. These accounts constitute a distinct analytic register, operating at the threshold where continuation itself is at risk.

Support was described as immediate, embodied, and interruptive. Animals were described as disrupting trajectories of harm through presence, proximity, and behavioural insistence. In several accounts, animals physically intervened—approaching, vocalising, demanding attention, or refusing to leave—thereby breaking dissociative states or narrowing attention away from self-destructive impulses:

“In an earlier attempt at suicide, my first cat walked in and snapped me out of the dissociation I was experiencing at the time. I remember ‘waking up’ with a knife to my wrists sobbing heavily. This is the exact reason I still have cats today.”

*(Love, 2021, p. 180) (*27)

Survival-oriented support did not depend on participants’ capacity to articulate distress or seek help. Crisis moments were frequently described as involving withdrawal, cognitive narrowing, or an inability to communicate. In this context, animals served as non-verbal responders, engaging without requiring explanation, disclosure, or compliance—a feature that distinguishes companion animal support from many formal crisis interventions.

Responsibility assumed heightened significance under conditions of acute risk. Whereas earlier sections describe responsibility as sustaining daily action or identity, during a crisis, it functions as a moral boundary against self-harm or death. Participants described being unable to proceed with self-destructive acts because of anticipated consequences for their animals, particularly abandonment, distress, or neglect:

“At my worst point, when I began having suicidal ideation, the thought of abandoning my pets was unacceptable to me so I knew I had to stick around to care for them.”

*(Hawkins* et al.*, 2021, p. 552) (*31)

These survival mechanisms were not described as curative. Participants did not report that animals resolved suicidal ideation or eliminated distress. Instead, animals enabled individuals to remain alive during moments of peak risk, allowing distress to pass without escalation. Survival was situational and temporal, achieved through interruption rather than transformation.

### 3.6 Social connection, belonging and re-entry into the social world

Participants described how relationships with companion animals facilitated connections that extended beyond companionship alone. Animals served as social mediators, enabling contact, participation, and belonging in ways that were perceived as safer and more manageable than direct interpersonal engagement.

Animals provided a bridge between private distress and public presence. In contrast to social situations characterised by scrutiny, misunderstanding, or anticipated judgment, interactions mediated by animals were described as simpler and more predictable. Walking a dog, caring for an animal in shared spaces, or being encountered in relation to a pet allowed participants to be socially visible without their mental health status becoming the primary frame through which they were understood:

“You’re just walking a dog and that’s kind of all they see about you [ … ] there’s not kind of like barrier up because, oh, you’ve got a mental health problem.”

*(Brooks* et al.*, 2019, p. 330) (*33)

Companion animals also supported social connection through non-demanding companionship that mitigated loneliness without requiring disclosure or emotional labour, or conversational reciprocity. Participants described animals as present, attentive, and available, allowing connection to be experienced without the pressures commonly associated with human relationships:

“Sometimes you just need a bit of comfort, and you want it silent [ … ] and you can do that a lot easier with a dog than with a person.”

*(Sudbury-Riley, 2024, p. 1934) (*34)

Socially mediated support extended to casual encounters in public spaces, structured group activities (e.g., training or walking groups), or renewed contact with family members around shared care practices. These forms of engagement were typically incremental and low-risk, allowing participants to remain socially involved without intensity or exposure.

### 3.7 Boundaries, loss and the limits of support

Participants’ accounts also delineated clear boundaries and limits to companion animal support. Relationships with companion animals, while deeply meaningful, were not without cost, vulnerability, or constraint.

Loss and fragility were salient boundaries. The death, illness, or anticipated loss of a companion animal was profoundly destabilising, particularly where the animal had been central to routine, responsibility, or identity. Loss was experienced not only as emotional bereavement but as the sudden withdrawal of structuring mechanisms that had previously sustained daily life and selfhood. Participants described losing a sense of responsibility, purpose, or orientation following the death of an animal, underscoring the extent to which these mechanisms were relationally anchored rather than internally held:

“She obviously needed me to look after her [ … ] I kind of lost that responsibility [when she died].”

*(Brooks* et al.*, 2019, p. 330) (*33)

Participants also described material and practical constraints, including financial strain, housing insecurity, and restricted access to services or accommodation. While responsibility for animals was often described as sustaining and morally meaningful, these obligations could also generate stress, guilt, or conflict, particularly in contexts of limited resources or unstable living conditions. Such accounts highlight that responsibility, while protective, could intensify vulnerability when structural supports were absent:

“I had to downsize my life. My life took a sudden change and I had to make a decision because he was important to me. And I wouldn’t just move anywhere. They had to take [dog] too.”

*(Kabel* et al.*, 2015, p. 412) (*35)

Participants did not present companion animals as sufficient substitutes for human or professional support. Several accounts explicitly acknowledged that animals could not address all dimensions of distress, particularly in relation to trauma processing, practical assistance, or sustained crisis care. Animals were described as providing comfort, containment, and continuity, but not resolving underlying social, economic, or structural problems. This reinforces companion animal support as complementary rather than comprehensive, operating alongside formal mental health services.

Finally, participants reflected on the conditional nature of access to companion animal support. Housing policies, institutional rules, and socioeconomic constraints shaped who could keep animals and under what conditions, positioning companion animal support as unevenly distributed and raising questions about equity and accessibility.

### 3.8 Mechanisms extending beyond the therapeutic

The synthesis identified several mechanisms through which companion animals provided support that formal therapeutic provision is structurally constrained from delivering. These are not framed as evidence of therapeutic superiority, but as forms of support that occupy distinct relational and temporal positions unavailable within professionalised care.

Six domains of divergence were identified. First, physical touch and tactile comfort: embodied co-regulation through stroking, warmth, and proximity that reduces arousal without cognitive work—most talk therapies prohibit or constrain sustained tactile contact. Second, constancy without discharge: day-to-day co-presence and relational continuity across relapse, withdrawal, and service transitions—therapeutic relationships are time-limited, sessional, and contingent on eligibility. Third, non-contingent acceptance: affective safety rooted in perceived unconditional acceptance and low evaluative threat—therapies aim for non-judgement but remain evaluative through monitoring, goals, and risk-management. Fourth, non-verbal presence and low demand: support delivered through quiet co-presence without requiring articulation, insight, or performative progress—conventional psychotherapy assumes verbalisation and reflective work. Fifth, authenticity and mutuality: perceived genuineness arising from a non-contractual bond—professional ethics require asymmetry and boundaries that restrict real-world reciprocity. Sixth, availability during crisis and service gaps: immediate access to support during acute distress and outside service hours—scheduled care cannot guarantee in-the-moment support, whereas pets are co-located. See **Supplementary Material 4** for full analysis.

### 3.9 Summary of synthesised mechanisms

The findings suggest that support from companion animals functions through a layered, relational process rather than as isolated effects. Fundamental mechanisms like emotional co-regulation, safety, and trust create the relational conditions necessary for other types of support to emerge. These conditions facilitate mechanisms that structure daily life and shift agency through routine and responsibility, thereby aiding in the reconstruction of identity, meaning, and existential orientation.

These mechanisms are neither purely linear nor uniform. They interact in a dynamic way, with later mechanisms providing feedback to stabilize earlier ones, and their prominence changing under different stress conditions. During intense psychological crises, multiple mechanisms combine into a survival-focused response characterized by immediacy, interruption, and moral restraint, rather than reflection or insight. In addition to formal therapeutic methods, the synthesis also highlights support domains like tactile comfort, consistency, unconditional acceptance, and crisis support—areas that professional care often cannot fully address structurally.

**Figure 2:**
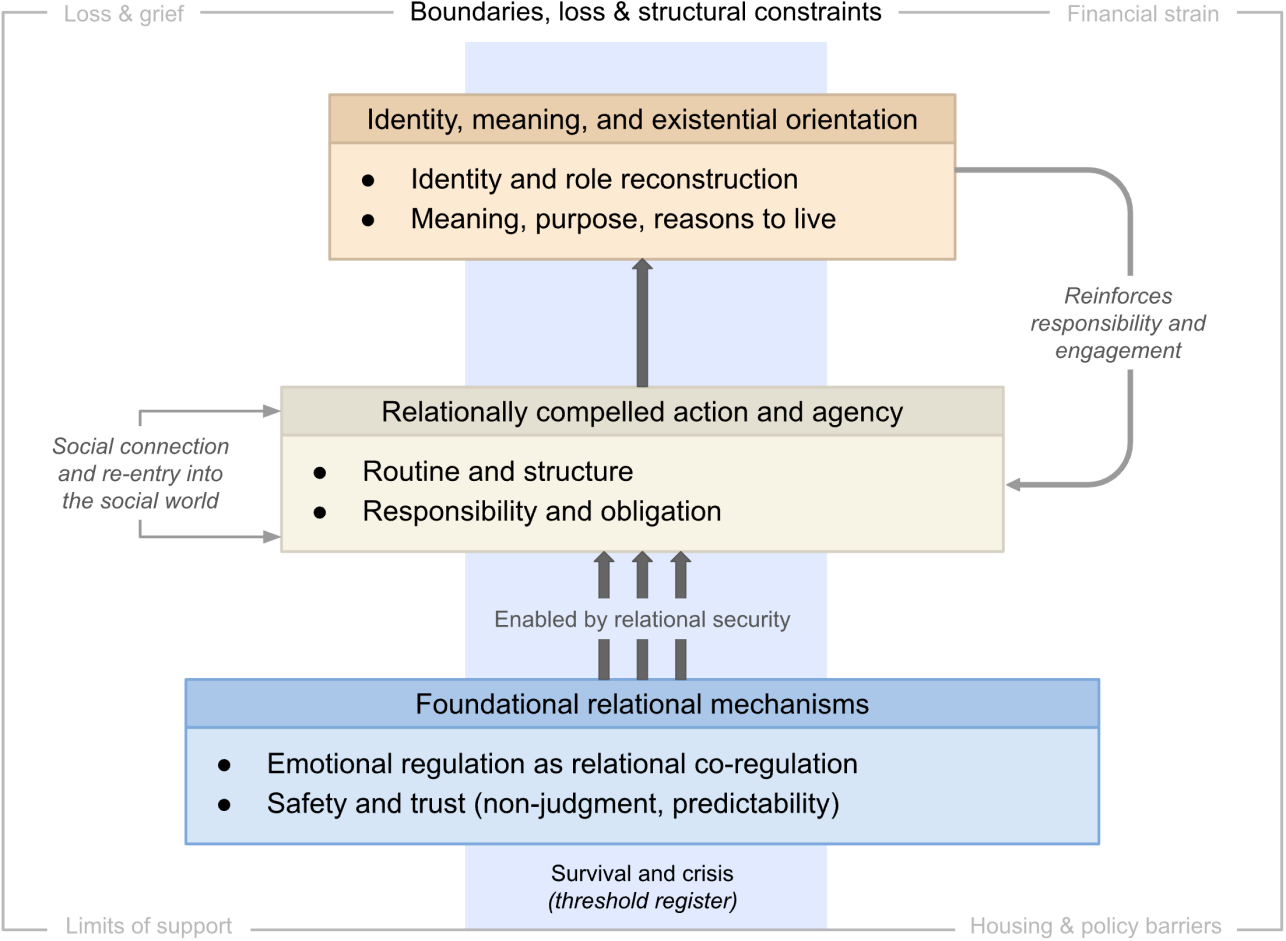
Conceptual synthesis of the layered relational mechanisms through which companion animals support mental health. This figure illustrates how companion animals support mental health through interconnected, non-linear mechanisms across distress, crisis, and structural constraints. Core relational mechanisms—emotional regulation, safety, and trust—operate at a survival threshold to establish security. This security fosters action and agency driven by relational commitment rather than deliberate effort, facilitating identity reconstruction, meaning, and reasons to live. Feedback loops reinforce ongoing responsibility and engagement. Social connection and reintegration are shown as contingent pathways, with boundaries, loss, and structural barriers framing all mechanisms, emphasising that support is partial, context-dependent, and not universally protective.

**Figure 2** illustrates this multilevel process, depicting how foundational relational mechanisms enable emergent forms of action, identity, and meaning, while also highlighting the threshold conditions under which survival-oriented support becomes critical. The figure situates these processes within the broader boundaries of loss, dependency, and structural constraint, underscoring both the generative potential and the inherent limits of companion animal support for mental health.

## 4 Discussion

This review examines the therapeutic mechanisms of companion animals in the context of previous literature on the mental health benefits of pets. Previous literature has pointed to pets’ contributions to social connection and emotional support, with some suggesting that, at times, people obtain more emotional support from pets than from some health professionals (7). The aim of the review here was to explore and identify specific therapeutic mechanisms that cast the role of companion animals as potential therapeutic agents operating in open systems of everyday life. Such mechanisms implicitly coexist in comparative terms of input alongside mental health professionals operating within more formalised mental health therapeutic traditions. The findings not only show overlap and similarities between the two in terms of rendering psychological benefits but also their uniqueness. The latter includes mechanisms of emotional regulation and sustained relational presence, which are less readily provided via conventional therapist-led models of therapeutic input.

The conceptual model developed in this review clarifies how relational security, regulation, agency, and meaning are sequenced in participants’ accounts. Attachment and social buffering perspectives help explain why animals’ perceived availability and non-judgment may be regulating, because emotionally salient relationships can reduce distress through felt security, proximity, and perceived support (10, 11). However, the synthesis suggests that regulation alone is insufficient to capture the therapeutic work described across studies. Companion animals also reorganised time, obligation, and self-understanding through repeated practices of care, extending Brown’s account of companion animals as relational figures that may support emotional containment, self-continuity, and felt recognition (12). In this respect, the findings align with relational approaches to human-animal wellbeing, where benefit emerges through shared routines and interdependence rather than from ownership as a static exposure (13). The model, therefore, reframes companion animals as everyday relational agents through which recognised therapeutic processes are enacted outside formal provision, while also showing why these processes are not reducible to existing therapy models.

The findings of this review do not indicate that pets can replicate or replace the full gamut of formalised therapeutic interventions provided by formal services. For example, companion animals were not described as meeting needs that require emergency risk management, or as therapies that rely on intensive interpretation work, specialised trauma processes, or techniques (e.g., EMDR, exposure therapy, cognitive restructuring). However, the synthesis did identify powerful, and in some cases more effective, contributions that pets made to core therapeutic mechanisms. For example, companion animals were often described as helping people to resist or as directly interrupting risky actions at the moment of heightened danger, demonstrating a salient protective effect in certain circumstances. Certainly, companion animals function as much more than a parallel or complementary form of formalised psychological aid, but rather as a form that operates outside institutional settings, aligning closely with everyday lived experience. Firstly, of note, is availability through presence and continuity (8). Contrary to access to formalised sources, which help with what is obtained from pets, there are no waiting lists, institutional gatekeeping, the need to fit within restrictive appointment systems, or time-limited or rationed numbers of sessions. These barriers are particularly pronounced for marginalised groups, exacerbating health inequalities; however, access to pet ownership is itself shaped by socioeconomic factors, including housing security and financial resources (35). Companion animals provide just-in-time, constant support without having to contend with these barriers. Pets have the capacity to always provide regulation and reassurance - including in moments when formal provision is unattainable or unavailable.

Second, an important mechanism is companion animals’ ability to offer non-evaluative companionship (36). Unlike formal therapeutic encounters, which are embedded in asymmetrical power relations and professional assessment, human–animal relationships tend to be free of judgment, blame, or performative expectations (37, 38). This may reduce the stress associated with self-monitoring, defensiveness, and fear of misinterpretation, which can impede seeking formal help and the development of a therapeutic alliance. For those with experiences of past contact with mental health services which have been negative or coercive, the absence of evaluative scrutiny may be particularly therapeutic.

Third, pets facilitate behavioural regulation through routines, touch, and shared activities. Daily pet-focused tasks demand structure, encourage physical exercise, and prioritise attention in the present (33). These are akin to, but not identical to, those of behavioural activation and mindfulness-based approaches. Rather, they are achieved naturally in the moment rather than through formalised intervention. Such attributes may be particularly relevant for individuals who do not wish to engage with more abstract cognitive strategies or approaches that demand insight and self-reflection (39).

Fourthly, mechanisms generated through encounters with companion animals may counter some of the limitations and risks associated with professional encounters. Psychotherapeutic outcomes vary substantially at an individual level, with formal therapy sometimes employing mismatched approaches (40), risking boundary violations and leading to general feelings of invalidation (41). Within this context, the advantages of pets lie in their ability to provide a sense of predictability, emotional security, and relational safety. Pets simplify rather than complicate interactions and offer unconditional consistency. They are unable to reinterpret narratives, impose rigid theoretical frameworks, or withdraw care based on insight, adherence, therapeutic progress, or transference issues. Thus, even when there are clear theoretical similarities with features of formal therapeutic approaches such as unconditional positive regard, formal provision is bounded by session limitations and delivered as therapeutic techniques rather than enacted as an ongoing, continually available presence.

The review findings illustrated how relationships with companion animals can operate both preventively and therapeutically. What individual studies did not show to the same extent, but which remains an important contextual factor, is the growing preference for informal sources of mental health support, such as companion animals, over professional input. This is likely to reflect broader structural pressures in mental health services, including rising costs of care, fragmented service provision, long waiting lists and declining trust in professional authority (42). In this sense, pets can be understood not merely as therapeutic supports, but also as indicators of unmet needs within mental health systems. Their perceived effectiveness highlights the value of continuity, relational simplicity, and non-instrumental care—qualities that are increasingly difficult to obtain and sustain within contemporary mental health provision.

Future research would benefit from moving beyond binary comparisons of pets versus therapists, toward models that examine how informal, non-human support systems interact with formal care over time. Longitudinal and mixed-methods studies could clarify which populations derive the greatest benefit from companion animals, under what conditions pets may substitute for or delay professional help-seeking, and how mental health services might integrate or acknowledge these forms of support without co-opting or medicalising them.

The review should be interpreted in light of its narrative and interpretive design. Although the updated Google Scholar search enabled identification of relevant qualitative studies across a dispersed interdisciplinary literature, the update did not rerun all databases used in the earlier systematic review. The synthesis, therefore, prioritises conceptual depth and theoretical integration over exhaustive evidence surveillance. Future systematic reviews could usefully update the full database search strategy to examine whether additional studies alter the evidential coverage of the mechanisms identified here.

Taken together, the therapeutic value of pets lies not in seeing them as simply replicating formal mental health provision, but in offering an alternative relational ecology—one characterised by constancy, non-judgement, and embodied co-regulation. Recognising these challenges, dominant assumptions about where and how mental health support must occur, and invites a broader, more pluralistic understanding of care.

### 4.1 Implications for mental health policy and service delivery

This review suggests that the perceived therapeutic value of companion animals cannot be fully understood as a matter of individual preference or as an informal alternative to professional mental health care or peer support. Rather, it reflects broader structural conditions within contemporary services, particularly those related to access, continuity, and trust. Therefore, the growing appeal of pets as therapeutic assets should not be understood as a rejection of formal therapy itself, but rather as a response to the institutional constraints that shape when, how, and for whom professional care is available.

A defining contrast between companion animals and formal mental health provision lies in the temporal availability of each. Mental health provision globally is characterised by stepped care models, short-term interventions, and rationed access based on diagnostic thresholds. While such models may be defensible from a resource-allocation perspective, they leave large populations with ongoing, fluctuating, or subclinical distress without sustained support. Pets, by contrast, provide continuous, low-intensity, just-in-time regulation, addressing the gaps that contemporaneous service models structurally produce.

From a policy standpoint, this raises questions about the implicit assumptions underpinning current service design—namely, that therapeutic benefit is primarily delivered through time-limited, professionalised encounters, rather than through ongoing relational and environmental stability. The evidence reviewed here suggests that for many individuals, especially those experiencing loneliness, chronic stress, or marginalisation, continuity and presence may be as relevant as therapeutic technique or modality.

The findings also point to matters of institutional trust and relational asymmetry. While psychotherapy is regulated and evidence-based, service users’ experiences are shaped not only by outcomes but by power dynamics, gatekeeping, and structures of accountability (42). Perceived invalidation, cultural mismatch, or, in some cases, abuse within therapeutic settings—albeit not representative of all practice—have a disproportionate impact on trust in services (41, 43). Companion animals offer a form of support that is structurally non-coercive and non-evaluative, which may be particularly salient in contexts where service users feel scrutinised or assessed rather than supported.

This has implications for how policy frameworks conceptualise “engagement” and “non-engagement.” Disengagement from formal services is often framed as individual non-compliance, yet the preference for pets as a primary source of emotional support suggests a more rational adaptation to systems perceived as costly, episodic, or emotionally unsafe. In this sense, companion animals may function as informal infrastructures of care, compensating for gaps left by formal provision rather than undermining it.

Economics also plays a critical role. In the UK and many European countries, access to high-quality psychotherapy often depends on private payment, while publicly funded services are overstretched. Pets, despite their own costs, represent a non-marketised form of care once acquired, offering ongoing benefits without limits on the number of sessions or eligibility criteria. This raises policy questions about equity: those unable to access sustained professional support may be relying on animals not out of preference, but necessity.

From a systems perspective, the therapeutic effects associated with pets—routine, embodied interaction, responsibility, and social buffering—closely resemble elements of interventions otherwise delivered through formal provision (e.g., behavioural activation, social prescribing). Yet policy approaches often seek to professionalise or instrumentalise these elements, rather than recognising the value of their emergence in naturally occurring relationships. This raises an important consideration for policymakers: without careful framing, attempts to integrate animals into service delivery risk undermining the very relational qualities that make these forms of support effective in the first place.

For policymakers, the implications are not that pets should replace therapists, but that mental health systems may be over-reliant on professionalised, episodic care while under-valuing continuous, relational, and embedded forms of support in daily life. Recognising companion animals as part of the broader mental health ecology challenges dominant hierarchies of care and invites reconsideration of how support is distributed, funded, and legitimised. Future policy-relevant research should therefore examine how informal supports, particularly non-human ones, interact with formal services over time, and how systems might be designed to reduce reliance on crisis-driven interventions by strengthening everyday sources of stability. Importantly, this should be done without shifting responsibility for mental health from institutions to individuals or their pets.

Overall, the preference for pets over therapists among some populations should be interpreted as a signal of systemic strain rather than a rejection of professional care. Companion animals illuminate what current mental health systems struggle to provide: continuity, non-judgement, and presence. Addressing this gap requires not only clinical innovation but policy frameworks that take relational and environmental supports seriously as components of public mental health.

### 4.2 Conclusion

A mechanisms-based perspective reveals that the perceived therapeutic advantage of companion animals arises not from superior efficacy, but from structural alignment with everyday human needs that formal services struggle to meet. Pets make visible the limits of professionalised, episodic care and highlight the importance of continuity, non-judgement, and embodied presence. For UK and European mental health policy, this represents an opportunity not to replace therapy, but to reconsider the institutional conditions under which therapeutic mechanisms can meaningfully operate.
