# Supplementary material for: Companion animals and mental health: a narrative synthesis of how pets deliver therapeutic mechanisms outside formal provision
Source: Front Psychiatry. 2026 Jul 1;17:1803383. doi: 10.3389/fpsyt.2026.1803383 (PMC13370905; doi:10.3389/fpsyt.2026.1803383)
Supplement: Supplementary file 1 [file Table1.docx]

# Supplementary materials

## Supplementary material 1

**Table S1**

Search terms

| Search area | Terms |
| --- | --- |
| Area 1: Pet ownership | “pets”; “companion animal*”; “domestic animal*”; “household animal*”; “pet ownership”; “animal-assisted” OR “animal-assisted therapy”; “emotional support animal*” |
| Area 2: Diagnosed mental health conditions or co-morbid mental health related to long-term physical conditions | “mental health”; “mental illness”; “mental disorder*”; “psychiatric illness” OR “psychiatric health”; “mental distress” OR “mental difficulty*”; depression OR anxiety OR PTSD OR “post-traumatic stress” OR bipolar OR schizophrenia; “chronic illness*”; “chronic condition*”; “long term condition*”; “comorbid*” OR “co-morbid*” |
| Area 3: Qualitative research terms | qualitative OR “qualitative research”; “interview*” OR “focus group*”; “thematic analysis”; “grounded theory”; “ethnograph*” OR narrative; “content analysis”; “interpretative phenomenological analysis” OR IPA |

Note. The April 2025 update used the same core pet ownership and mental health search concepts as the original systematic review (8), with the addition of qualitative research terms to focus retrieval on studies reporting qualitative data. Terms were used iteratively and in combination in Google Scholar rather than as a single fixed database string.

## Supplementary material 2

Analysis guide

**Overview**

This document outlines the analytical framework used to conduct the narrative synthesis. The analysis proceeded through three main phases: clustering mechanisms from the extracted data, mapping these onto formal therapeutic mechanisms, and identifying where companion animals provide support that formal therapy cannot. These phases correspond to the three results sections of the article.

**Phase 1: Mechanism Clustering**

The first author (EV) created a working spreadsheet from the data extraction file with columns for Study ID, Original Mechanism Text, Cluster Assignment, and Notes. Each discrete mechanism statement was entered as a separate row. EV read through all mechanism statements without coding initially, noting recurring concepts. Provisional clusters emerged including: presence and companionship; emotional regulation; routine and structure; responsibility and obligation; identity and role; meaning and reason to live; safety and trust; social mediation; and sensory/tactile mechanisms. EV then worked through each mechanism statement systematically, assigning it to one or more clusters, noting secondary relevance where statements spanned multiple clusters, and creating new provisional clusters where needed. Once coding was complete, EV created a cluster summary table showing each cluster name and definition, the number of contributing studies, the populations represented, and illustrative quotes.

**Phase 2: Mapping onto Formal Therapeutic Mechanisms**

EV selected focal therapies where the mapping was most substantive and evidenced in the data: Behavioural Activation, Attachment-based therapy, Compassion-Focused Therapy, DBT (distress tolerance), Existential therapy, Humanistic/person-centred approaches, Mindfulness-based approaches, and Trauma-informed approaches. EV created a mapping matrix with mechanism clusters as rows and focal therapies as columns, indicating mapping strength and the nature of each pairing. For each cluster-therapy pairing, EV considered whether the relationship constituted approximation (pet relationship mimics the therapy mechanism imperfectly), instantiation (pet relationship enacts the same mechanism through different means), or something fundamentally distinct despite superficial resemblance.

**Phase 3: Identifying What Pets Provide That Therapy Cannot**

EV returned to the mechanism statements and supporting quotes, specifically looking for explicit contrasts with human or professional relationships, mechanisms with no clear therapy equivalent, and mechanisms where therapy approximates but with constraints. From this review, EV developed gap categories including: physical touch and tactile comfort; constancy without discharge; non-contingent acceptance; non-verbal presence; authenticity and mutuality; moral obligation to another; and availability during crisis. EV paid particular attention to data about suicide prevention, reasons to live, and survival, examining how pets sustain life when hope, insight, or recovery-oriented progress are absent.

**Quality Considerations**

EV kept reflexive notes throughout the analysis, documenting interpretive choices, what was foregrounded and backgrounded, and the theoretical commitments shaping the first author’s reading of the data. Working spreadsheets including the mechanism coding and mapping matrix are available on request. Every claim in the narrative is traceable to specific studies and quotes, and where evidence was thin the authors acknowledged this limitation.

**Outputs**

The analysis produced: a mechanism coding spreadsheet with all mechanism statements assigned to clusters; a cluster summary table with definitions, study counts, populations, and illustrative quotes; a mapping matrix showing mechanism clusters against focal therapies; and a list of gap categories with supporting quotes.

## Supplementary material 3

**Table S2**

Study-by-mechanism contribution matrix

|  | Mechanism number | | | | | | | | | | |  |
| --- | --- | --- | --- | --- | --- | --- | --- | --- | --- | --- | --- | --- |
| Study ID | 1 | 2 | 3 | 4 | 5 | 6 | 7 | 8 | 9 | 10 | 11 | Total |
| Brooks et al. (2019) | ✓ | ✓ | ✓ |  | ✓ | ✓ |  | ✓ |  |  |  | 6 |
| Schmitz et al. (2022) | ✓ | ✓ | ✓ |  | ✓ | ✓ |  |  |  |  |  | 5 |
| Young et al. (2020) | ✓ |  | ✓ |  | ✓ | ✓ | ✓ |  |  |  |  | 5 |
| Scanlon et al. (2021) | ✓ |  | ✓ |  | ✓ | ✓ | ✓ | ✓ |  | ✓ |  | 7 |
| Fossey et al. (2020) | ✓ | ✓ | ✓ |  | ✓ |  |  |  |  |  |  | 4 |
| Kosteniuk & Dell (2020) | ✓ | ✓ | ✓ |  | ✓ | ✓ | ✓ |  |  |  |  | 6 |
| Love (2021) | ✓ | ✓ |  |  | ✓ | ✓ |  | ✓ |  |  |  | 5 |
| Brooks et al. (2016) | ✓ | ✓ | ✓ |  | ✓ |  |  | ✓ |  |  |  | 5 |
| Gaughan (2021) | ✓ | ✓ | ✓ |  |  | ✓ |  |  |  | ✓ |  | 5 |
| Williamson et al. (2022) | ✓ | ✓ | ✓ |  |  | ✓ |  |  |  |  | ✓ | 5 |
| Hawkins et al. (2024) | ✓ | ✓ | ✓ | ✓ |  |  |  |  |  | ✓ |  | 5 |
| Barcelos et al. (2021) | ✓ | ✓ | ✓ | ✓ | ✓ |  |  |  |  |  |  | 5 |
| Wisdom et al. (2010) | ✓ | ✓ | ✓ | ✓ | ✓ |  |  | ✓ |  |  |  | 6 |
| Zimolag (2017) | ✓ | ✓ | ✓ |  | ✓ |  | ✓ |  |  |  |  | 5 |
| Schmitz et al. (2021) |  | ✓ | ✓ |  |  | ✓ |  |  | ✓ | ✓ |  | 5 |
| von Humboldt et al. (2024) | ✓ | ✓ | ✓ |  |  | ✓ |  |  |  |  |  | 4 |
| Zablan et al. (2023) | ✓ | ✓ | ✓ |  |  | ✓ |  |  |  |  |  | 4 |
| Gan et al. (2020) | ✓ | ✓ | ✓ |  |  | ✓ |  |  |  |  |  | 4 |
| Cyr & Hawkins (2024) | ✓ | ✓ | ✓ |  |  | ✓ |  |  |  |  |  | 4 |
| Garland-Lewis et al. (2024) | ✓ | ✓ | ✓ | ✓ | ✓ |  |  |  |  |  |  | 5 |
| Hawkins et al. (2021) | ✓ | ✓ | ✓ |  |  | ✓ |  |  |  |  |  | 4 |
| Kerr-Little et al. (2023) | ✓ | ✓ | ✓ | ✓ | ✓ |  |  |  |  | ✓ |  | 6 |
| Kabel et al. (2015) | ✓ | ✓ | ✓ |  |  | ✓ |  |  |  |  |  | 4 |
| Sudbury-Riley (2024) | ✓ | ✓ | ✓ |  |  | ✓ |  |  |  |  |  | 4 |

Note. 1 = Emotional regulation; 2 = Safety and trust; 3 = Routine and structure; 4 = Responsibility and obligation; 5 = Identity and role; 6 = Meaning and reason to live; 7 = Presence and companionship; 8 = Social mediation; 9 = Sensory/tactile; 10 = Practical support and access; 11 = Unclear/other. Blank cells indicate that the mechanism was not coded as a primary mechanism cluster for that study; absence of a tick does not indicate contradictory evidence. Matrix based on primary cluster assignment only.

## Supplementary material 4

Mechanisms beyond the therapeutic analysis table

| Gap category | Definition (analytic) | Primary clusters implicated | Studies with evidence | Quote examples |
| --- | --- | --- | --- | --- |
| Physical touch and tactile comfort | Embodied co-regulation via stroking, warmth, proximity, and sensory grounding that reduces arousal without cognitive work. | Emotional regulation, Identity and role, Meaning and reason to live, Practical support and access, Responsibility and obligation, Routine and structure, Safety and trust, Sensory/tactile | 5 | “So we cuddle … petting him and cuddling with him and having him close to me … just knowing that he’s with me.” (Participant 2; dog; p. 66) “She comes over like that, and then lies on me. I find that the combination of stroking her and her lying on me it’s almost like having your personal kind of heated weighted blanket. It’s just really calming.” (P1; dog; p. 6) “I mean I call it ‘thera-purry’… lying on me and purring is like… way better than professional therapy I’ve gone to.” (Alex; cat; p. 9) |
| Constancy without discharge | Relational continuity characterised by day-to-day co-presence and endurance across relapse, withdrawal, and service transitions. | Emotional regulation, Identity and role, Meaning and reason to live, Practical support and access, Presence and companionship, Responsibility and obligation, Routine and structure, Safety and trust | 5 | “Having pets has always been a grounding rod for me… I kept [jobs] because they made it so I could make sure that the pets were safe.” (Avery; cats; p. 13) “You go to AA groups, you can’t say everything you want in those places… Somebody’s always opinionated… When you have a dog, and you can talk for an hour or two hours or whatever, you sit there and you’re with your animals.” (Michael; dogs; p. 96) “Having my cat close to me helped me feel calmer and less anxious, especially at night.” (Participant; cat; p. 7) |
| Non-contingent acceptance and non-judgement | Affective safety rooted in perceived unconditional acceptance, low evaluative threat, and diminished shame/stigma exposure. | Emotional regulation, Identity and role, Meaning and reason to live, Practical support and access, Presence and companionship, Responsibility and obligation, Routine and structure, Safety and trust, Sensory/tactile, Social mediation | 5 | “We come back to unconditional love, support, non-judgementalism. I won’t say it’s relaxing, it…it’s reassuring, it’s supportive, and it gives you a boost.” (ID3; two cats; p. 4) “Animals are so much more supportive to me than humans are, because humans judge. Animals don’t.” (Natalie; dog; p. 10) “In some ways it is nice because they don’t talk… they don’t nag… Sometimes people tend to put pressure on you… They just accept what you are that day.” (Abigail; cat; p. 96) |
| Non-verbal presence and low demand | Support delivered through quiet co-presence that does not require articulation, insight, or performative progress. | Emotional regulation, Identity and role, Meaning and reason to live, Routine and structure, Safety and trust, Social mediation | 2 | “He’d just sit there, exuding peace.” (ID6; one cat; p. 3) “When my wife died, she was consoling to me, without talking to each other… The recovery was a lot shorter because of the dog.” (Ben; dog; p. 4) |
| Authenticity and mutuality beyond professional boundaries | Perceived genuineness and reciprocity arising from a non-contractual bond, contrasted with time-limited, role-bound professional relationships. | Emotional regulation, Meaning and reason to live, Practical support and access, Routine and structure, Safety and trust, Sensory/tactile | 2 | “Bella is very active, so I really have a therapy session. She can cheer me up, and I love to be close to her … I now socialize and go to work.” (Participant 3; cat; p. 84) “I mean I call it ‘thera-purry’… lying on me and purring is like… way better than professional therapy I’ve gone to.” (Alex; cat; p. 9) |
| Moral obligation and reasons to live | Survival-oriented commitment generated by responsibility to a dependent other, sustaining life when hope or recovery orientation is absent. | Emotional regulation, Identity and role, Meaning and reason to live, Practical support and access, Presence and companionship, Routine and structure, Safety and trust, Sensory/tactile, Social mediation, Unclear/other | 5 | “Looking after her… she needed me to look after her… I kind of like lost that responsibility [when she died].” (ID8; one hamster; p. 6) “They give me a reason to live… I can’t be that person when I’m addicted… They give me a reason to live, for them.” (Robert; dogs; p. 97) “They give me a reason to live… you are not going to care for a dog [while using].” (Veteran 4; service dog; p. 31) |
| Availability during crisis and service gaps | Immediate access to support during acute distress and outside service hours, including nocturnal or high-risk moments. | Emotional regulation, Identity and role, Meaning and reason to live, Practical support and access, Presence and companionship, Responsibility and obligation, Routine and structure, Safety and trust, Sensory/tactile, Social mediation, Unclear/other | 6 | “Pets can help me feel happier… playing with pets kind of distracts my mind from bad thoughts such as suicide, self-harm, fighting.” (Lilly; pet not specified; p. 14) “Thoughts of self-harm… since I’ve had [dog’s name]… that’s where he comes in.” (Participant 17; dog; p. 10) “Well, we are truly a team right. I couldn’t imagine life without her, I really couldn’t.” (Veteran 4; service dog; p. 27) |
